# Optimization of fermentation conditions through response surface methodology for enhanced antibacterial metabolite production by *Streptomyces* sp. 1-14 from cassava rhizosphere

**DOI:** 10.1371/journal.pone.0206497

**Published:** 2018-11-14

**Authors:** Tian Yan Yun, Ren Jun Feng, Deng Bo Zhou, Yue Yun Pan, Yu Feng Chen, Fei Wang, Li Yan Yin, Yin Dong Zhang, Jiang Hui Xie

**Affiliations:** 1 Institute of Tropical Agriculture and Forestry, Hainan University, Haikou, P.R. China; 2 Key Laboratory of Biology and Genetic Resources of Tropical Crops, Ministry of Agriculture, Institute of Tropical Bioscience and Biotechnology, Chinese Academy of Tropical Agricultural Sciences (CATAS), Haikou, P.R. China; Tallinn University of Technology, ESTONIA

## Abstract

*Streptomyces* species 1–14 isolated from cassava rhizosphere soil were evaluated for their antibacterial efficacy against *Fusarium oxysporum* f.sp. *cubense* race 4 (FOC4). Of the 63 strains tested, thirteen exhibited potent antibacterial properties and were further screened against eight fungal pathogens. The strain that showed maximum inhibition against all of the test pathogens was identified by 16S rDNA sequencing as *Streptomyces* sp. 1–14, was selected for further studies. Through the propagation of *Streptomyces* sp. 1–14 in soil under simulated conditions, we found that FOC4 did not significantly influence the multiplication and survival of *Streptomyces* sp. 1–14, while indigenous microorganisms in the soil did significantly influence *Streptomyces* sp. 1–14 populations. To achieve maximum metabolite production, the growth of *Streptomyces* 1–14 was optimized through response surface methodology employing Plackett-Burman design, path of steepest ascent determinations and Box-Behnken design. The final optimized fermentation conditions (g/L) included: glucose, 38.877; CaCl_2_•2H_2_O, 0.161; temperature, 29.97°C; and inoculation amount, 8.93%. This optimization resulted in an antibacterial activity of 56.13% against FOC4, which was 12.33% higher than that before optimization (43.80%). The results obtained using response surface methodology to optimize the fermentation medium had a significant effect on the production of bioactive metabolites by *Streptomyces* sp. 1–14. Moreover, during fermentation and storage, pH, light, storage temperature, etc., must be closely monitored to reduce the formation of fermentation products with reduced antibacterial activity. This method is useful for further investigations of the production of anti-FOC4 substances, and could be used to develop bio-control agents to suppress or control banana fusarium wilt.

## Introduction

*Fusarium oxysporum* f. sp. *cubense* (FOC) is a soil-borne fungus that causes fusarium wilt, which is considered to be the most destructive disease of bananas [1]. Banana fusarium wilt occurs in all countries, mainly in the tropical banana-producing areas of Asia, Australia, Africa and America. No effective chemical agents for the prevention and treatment of this disease exist, thus, researchers want to use biological methods to control banana fusarium wilt [1–4]. Microbes are one of the most productive sources of natural products from which antibiotics are derived [5]. According to reports, 70% of active antimicrobial substances are produced by actinomycetes, and *streptomycetes* provide nearly 80% of all the antibiotics in the world. *Streptomyces* species are renowned sources of novel secondary metabolites that have a range of biological activities such as antimicrobial, anticancer, and immunosuppressive activities [6]. Such *Streptomyces strains* are continuously explored for antimicrobial drug discovery. Thus, new antibiotics may act through new mechanisms that could suppress banana fusarium wilt [7–8].

The production of secondary metabolites by microorganisms is highly dependent on the strains and species present as well as their nutritional and cultural conditions [6]. Minor changes in media composition can substantially impact the quantity and quality of secondary metabolites along with the general metabolic profiles of microorganisms. The optimization of culture media is very complicated because any material that supports microbial growth can be a potential substrate [9]. Therefore, designing an appropriate medium and determining the conditions for cultivation are of prime importance for improving the antibiotic yield [10]. Response surface methodology (RSM) is an experimental method that is a useful tool to optimize the parameters of fermentation processes by applying mathematics and statistics [11–12]. The use of RSM to optimize fermentation conditions is mainly based on the following experiments [13]: 1) using the Plackett-Burman design to identify factors that significantly impact the fermentation process; 2) determining the path of steepest ascent to ascertain the approximate range of the best fermentation conditions using the key factors; and 3) performing the Box-Behnken test design to establish the fermentation model and determine the optimal fermentation conditions. RSM has been widely used to optimize microbial fermentation processes, and it can determine the impact of multiple factors [14]. Furthermore, RSM can be used to optimize fermentation conditions such that they meet the nutritional needs of a specific microorganism, thus avoiding the unnecessary addition of excess components in the culture medium [15]. Compared with other optimization methods, RSM requires fewer trials to calculate the numerous variables and their interactions [12]. The RSM approach has been adopted to improve the production of antibacterial compounds in several *Streptomyces* species, including *Streptomyces* sp. HJC-D1 [11], *Streptomyces nogalater* NIIST A30 [16], *Streptomyces* sp. SY-BS5 [17], and *Streptomyces* sp. SYYLWHS-1-4 [18]. Therefore, RSM strategies to maximize the yields of bioactive metabolites are necessary [19].

The objective of this study was to characterize the antagonistic actinomycete strain 1–14, which was identified based on its 16S rDNA sequence and phenotypic characteristics, and to optimize its fermentation conditions by RSM to enhance its antibacterial metabolite production. To date, no reports have examined actinomycetes from cassava rhizosphere soil. Investigating unstudied environments can enable the identification of new strains, which could, in turn, lead to the discovery of new compounds for use in new pesticides to protect plants, thus developing new resources. These methods can be used to develop bio-control agents to prevent and control banana fusarium wilt.

## Materials and methods

### Sample collection and actinomycete strain isolation

Cassava (*Manihot esculenta* Crantz) rhizosphere soil was collected in Haikou City, Hainan Province, China. No permissions were required for these collections in a campus area. This study did not involve any endangered species. The soil samples were collected in sterile bags. Each soil sample was air dried, thoroughly ground and sieved. Subsequently, 1 g of each soil sample was suspended in 10 mL of sterile distilled water and then incubated at 55°C for 20 min in a shaking incubator (Shanghai Zhicheng Company, China) at 180 rpm to prepare a suspension [20–21]. The resulting supernatants were diluted serially 10-fold to prepare and aliquot suspensions at 10^−1^, 10^−2^ and 10^−3^. Aliquots of 100 μL from each dilution were evenly spread plated on actinomycete isolation media (HV, SIM, SCA, and GA) in triplicate and incubated at 28°C for 2–4 weeks. Different single colonies were selected and purified on YE medium.

#### Pathogenic filamentous fungi

The pathogenic filamentous fungi used in this study included *Alternaria musae* (ATCC 66984), *Botrytis cinerea* Persoon (ATCC 11542), *Colletotrichum acutatum* Simmonds (ATCC 56815), *Colletotrichum fragariae* Brooks (ATCC 58718), *Colletotrichum gloeosporioides* (ATCC 16330), *Curvularia lunata* (Wakker) Boedijn (ATCC 60935), *Colletotrichum musae* (ATCC 96167) and *Fusarium oxysporum* f. sp. *Cubense* race 4 (ATCC 76255). The fungi were maintained on Petri plates containing potato dextrose agar (PDA) at 4°C and were subcultured every 2 months.

#### Antagonistic strains screening

The primary screening of 63 actinomycetes isolates was performed using the plate confrontation culture method. The actinomycetes strains were inoculated at a distance of 2.5 cm from the pathogen strains, with 4 pathogen strains inoculated per dish; identical dishes inoculated with FOC4 as the treatment strain were used as controls. Activity was recorded after 7 days of growth at 28°C. The colony growth diameters of the pathogens were measured by the cross method and the rate of inhibition of mycelial growth were calculated using the following formula [22]:

$$\mathrm{Antibacterial}\;\mathrm{rate}\;(\%)=\frac{A‑B}{A}\times 100$$

where A is the colony diameter of the control, and B is the colony diameter of the treatment.

In the secondary screening, 13 actinomycetes strains that exhibited significant activity against FOC4 were further tested against eight additional pathogens, to assess their broad-spectrum antibacterial properties. The strain with the best antibacterial rate was selected for further studies.

### Morphological, cultural and physiological characterization

Morphological, cultural, physiological, and biochemical characterization of the isolate was carried out as described by the International *Streptomyces* Project. The cultural traits of the strain were recorded on different media which including ISP 2 (Malt extract agar), ISP 3 (Oatmeal agar), ISP 4 (Inorganic salt starch agar), ISP 5 (Glycerol asparagine agar), ISP 6 (Peptone yeast extract agar) and ISP 7 (Tyrosine agar) [23–24].

The morphological characteristics of strain 1–14 were assessed by scanning electron microscopy (SEM). Sterilized cover slips were inserted into GA medium at an angle of approximately 45º, followed by culturing at 28°C for 8 days. Each cover slip sample was then coated with a film of gold under vacuum, and the mycelium and spore surface structures were viewed via scanning electron microscopy (model S-4800, Hitachi Limited, Japan).

Biochemical tests, including H_2_S production, nitrate reduction, urease activity, and cellulose, starch, and gelatine hydrolysis were also evaluated [25]. Physiological characterizations such as the effect of pH (4–10) and temperature (20–36°C), and NaCl tolerance were analyzed. Carbon sources utilization was determined in basal liquid medium (BLM) supplemented with various carbon sources at 1% (D-fructose, D-galactose, α-lactose, mannitol, soluble starch, xylose, rhamnose, arabinose, raffinose, melezitose, anhydrous lactose, D-trehalose, D-mannose, D-ribose, inositol, and sorbitol). Basal medium without a carbon source served as a control. Nitrogen utilization of the strain was examined in BLM with various nitrogen sources at 1% (ammonium sulphate, histidine, methionine, serine, oxamic acid, glycine, hydroxyproline, phenylalanine, glutamic acid, cysteine, arginine, valine, ammonium molybdate tetrahydrate, ammonium acetate, and ammonium nitrate). Basal medium without a nitrogen source served as a control.

### Molecular identification via 16S rDNA sequencing and phylogenetic analysis

Total genomic DNA was extracted from *Streptomyces* sp. 1–14 using a BioTek rapid genomic DNA extraction kit (DP1301, Beijing BioTek Biotech Co., Ltd., China). A 16S rDNA gene fragment was amplified using universal primers (forward primer 5’-AGAGTTTGATCCTGGC TCAG-3’ and reverse primer 5’-TACGGCTACCTTGTTACGACTT-3’) [26]. The amplification profile included an initial denaturation at 95°C for 5 min, followed by 35 amplification cycles of 94°C for 1 min, 55°C for 1 min and 72°C for 2 min. The PCR product was sent to Shanghai Sangon Biological Engineering Technology & Services Co., Ltd., for sequence determination. The sequence was compared to those of other related species downloaded from a public database using the EzBioCloud tool, and clustering analysis and phylogenetic tree construction using the Neighbor-Joining method were performed using MEGA 5.1 software [27–28].

### Propagation of *Streptomyces* sp. 1–14 in soil under simulated conditions

To prepare a *Streptomyces* spore suspension, spores of *Streptomyces* sp. 1–14 were gently eluted with 3 mL of sterile water, and added to sterile Erlenmeyer flasks, after which they were diluted to 0.8 × 10^8^ cfu/g. To prepare a suspension of FOC4, the pathogen was cultured for seven days. Subsequently, the mycelia and spores were eluted with 3 mL of sterile water into sterilized empty bottles, and sterile water was then added to 100 mL. Prior to use, this suspension was thoroughly mixed. To process the soil, 50 g of soil were weighed in 200 mL tissue culture flasks in pentaplicate. Two bottles of soil were not sterilized. The remaining three bottles were wet-sterilized at 121°C for 40 minutes once a day for two days, and one bottle was used to test whether the soil was completely sterilized.

The prepared soils were labelled as natural soil and sterile soil [29–30]. Two treatments for the different soil types were carried out: the inoculation of 5 mL of only the *Streptomyces* sp. 1–14 spore suspension in the natural and sterile soils and the inoculation of 5 mL of the *Streptomyces* sp. 1–14 spore suspension and 2 mL of the FOC4 suspension in the natural and sterile soils. Two millilitres of water were added to the soils every other day. The amount of *Streptomyces* sp. 1–14 in the soil was determined on the 7th, 14th and 21st days after inoculation. For this, soil samples (5 g) of each treatment were diluted serially 10-fold to dilutions of 10^−1^, 10^−2^ and 10^−3^ in triplicate. Aliquots of 50 μL from each dilution were evenly spread plated on GA medium (containing 80 μg/mL potassium dichromate), and the colonies were counted after 10 days of incubation at 28°C. The ratio of *Streptomyces* sp. 1–14 in the total number of soil strains was calculated using the following formula:

$$J(\%)=\frac{Q}{\sum A}\times 100$$

where *J* is the ratio of *Streptomyces* sp. 1–14 in the total number of soil strains, *Q* is the number *Streptomyces* sp. 1–14 spores inoculated into the soil, and ∑*A* is the total number of actinomycetes in the soil.

#### Optimizations of the experimental design for fermented crude extract and statistical analysis

RSM in our study included Plackett-Burman design, determination of the path of steepest ascent and BBD. To obtain fermented crude extract one hundred milliliters of the fermentation medium was dispensed into each 250 mL conical flask. Inoculated flasks were incubated for 8 days with shaking at 150 rpm and 28°C. Then, pure ethanol was added to the fermentation broth at a ratio of 1:1, and after mixing thoroughly, the flasks were allowed to stand for 10 days. Anti-FOC4 activity was determined using the filter-paper disc agar diffusion method [31].

**Plackett–Burman design.** Plackett-Burman design was employed to determine the most effective media composition using Design-Expert software (Version 10.0, Stat-Ease Inc., Minneapolis, USA) [32–33], it was performed using a fermentation culture medium at pH of 7.0 (g/L) (glucose, 30; soy flour, 20; MgSO_4,_ 0.2; NaH_2_PO_4,_ 0.5; CaCl_2_•2H_2_O, 0.1; and K_2_HPO_4,_ 0.5). Each independent variable was tested at two levels, high (+) and low (-). The levels of each factor are listed in S4 Table. A set of 12 experiments was conducted to evaluate the effects of the nine variables (S5 Table). Each row represents a trial, and each column represents an independent (assigned) or dummy (unassigned) variable. All experiments were conducted in triplicate. Statistical analysis of the model was performed to calculate the analysis of variance (ANOVA). Factors with a 5% level of significance (P<0.05) were considered to have a significant anti-FOC4 effect. The superiority of the polynomial model equation was judged by determining the coefficient R, and the statistical significance was defined by the F-test [11].

The data were analysed using SAS software, and the main factors influencing antibacterial activity were identified. Plackett–Burman experimental design is based on a first-order model:

$$Y=\beta_{0}+\sum \beta_{i}x_{i}$$

where *Y* is the response or dependent variable (antibacterial activity), *β*_*0*_ is the model intercept, *β*_*i*_ is the regression coefficient, and *x*_*i*_ is the independent variable.

**Determination of the path of steepest ascent.** According to the first regression equation obtained from the Plackett-Burman test results, the path of steepest ascent design was determined, and the step length and direction were calculated (Table 1). If the coefficient is negative, the level of the factor should decrease, and vice versa; thus, the larger the coefficient, the smaller the step size. In accordance with the design of the steepest ascent test design, the final ranges of the fermentation conditions needed to attain the best antibacterial activity of *Streptomyces* sp. 1–14 were determined.

**Table 1:** Experimental design and determination of the path of steepest ascent.

	Variable				Antibacterial activity (%)
	Glucose (g/L)	CaCl_2_·2H_2_O(g/L)	Temperature (°C)	The inoculation amount (%)	
(1) Test center point	33.75	0.12	34.50	11.25	
(2) Test step size	3.750	0.015	3.500	1.250	
(3) Slope	6.518	15.525	-5.635	-8.942	
(4) Corresponding value size = (2)×(3)	24.443	0.233	-19.723	-11.178	
(5) Step length = (4)×0.05^a^	1.222	0.012	-0.986	-0.559	
(6) Test No. 1	33.75	0.12	34	11.00	32.17
No. 2	34.97	0.13	33	10.5	35.12
No. 3	36.19	0.14	32	10.0	41.89
No. 4	37.41	0.15	31	9.5	46.31
No. 5	38.63	0.16	30	9.0	50.67
No. 6	39.85	0.17	29	8.5	43.23

Note: A representative factor of 0.05 is a factor determined by the experimenter based on process knowledge and other practical considerations that is appropriate in this example.

**Box-Behnken design and response surface analysis.** According to the principle of RSM, the best value derived for the factors from the path of steepest ascent determination is used as the center point of the response surface, and a response analysis of the key factors is then carried out [12, 34–35]. For a 3-level-3-factor BBD with three replicates at the center, a total of 29 experiments were performed to optimize the key factors (S6 Table). After the responses were measured for each trial, each response was fitted to an independent second-order polynomial model:

$$Y=\beta_{0}+\sum \beta_{i}x_{i}+\sum \beta_{ii}x_{i}x_{j}+\sum \beta_{ij}x_{i}x_{j}$$

where *Y* is the predicted response value (antibacterial rate), *β*_*0*_ is the constant term coefficient, *β*_*i*_ is the primary term coefficient, *β*_*ii*_ is the quadratic term coefficient, and *x*_*i*_ is the independent variable. The relationship between *x*_*i*_ and the true value of the independent variable *X*_*i*_ is the following:

$$x_{i}=\frac{X_{i}-X_{o}}{\delta X}$$

where *X*_*0*_ is the true value of the independent variable at the test centre point, and *δX* is the step change of the independent variable.

The statistical competence of the model was resolved through analysis of variance (ANOVA). F- and P-values were used to test the significance of each factor and regression model. If the F- and P-values of the regression model are large, the reliability of the regression model is high. The excellence of the polynomial model equation was determined statistically through the coefficient of determination (R^2^) and the adjusted R^2^. Three-dimensional response surface plots were produced to elucidate the relationships between the responses and the experimental levels of each independent variable. The optimum level of each variable for maximum antibacterial activity was resolved using the response optimizer tool of the software.

#### Studies on the stability of the crude fermentation extract

The stability of the crude fermentation extract was determined by investigating its antibacterial effect over time and after thermal, stored, pH and UV stress [36].

**Stability of the stored antibacterial.** Fifty-milliliter aliquots of the crude fermented extract were placed in sterile sealed bottles and stored at 4°C in a refrigerator or at room temperature (28°C). The anti-FOC4 activity of the aliquots was determined at 7, 14, 28, 49 and 77 d, with each treatment repeated in triplicate.

**pH stability of the antibacterial.** The pH of the crude extract was adjusted to 3, 4, 5, 6, 7, 8, 9 or 10 with 1 mol/L HCl or 1 mol/L NaOH. After the samples were allowed to stand for 2 h, the pH was adjusted back to its original value. Untreated extract at its original pH value was used as a control, and each treatment repeated in triplicate.

**UV stability of the antibacterial.** Crude extract was placed in sterile culture dishes and irradiated for 1, 3, 5, 7, 9 or 12 h using a UV lamp at a distance of 10 cm. Non-irradiated crude extract was used as a control, and each treatment repeated in triplicate.

**Thermal stability of the antibacterial.** Crude fermented extract was incubated in sterilized glass tubes for 60 min at 50°C, 60°C, 70°C, 80°C, 90°C or 100°C. Untreated fermentation broth was used as a control, and each treatment repeated in triplicate.

## Results

### Isolation of actinomycete strains and antagonistic strain screening

From the rhizosphere soil of cassava samples, 63 morphologically distinct actinomycete strains were isolated. Primary screening found 13 strains exhibited antibacterial activity against FOC4. Secondary screening was performed to ascertain the broad-spectrum antibacterial properties of these 13 actinomycetes strains. Strain 1–14 exhibited maximum antibacterial activity against all the test pathogens (Table 2; S1 Fig). Thus, strain 1–14 was selected for further studies.

**Table 2:** Inhibitory effects of strain 1–14 against 8 pathogens in dual cultures.

Test pathogen	Inhibition rate (%)
*Colletotrichum acutatum*	97.59%
*Colletotrichum gloeosporioides*	94.12%
*Botrytis cinerea*	92.31%
*Colletotrichum fragariae*	91.74%
*Curvularia lunata*	89.41%
*Fusarium oxysporum* f. sp. *Cubense* race 4	87.65%
*Colletotrichum musae*	79.41%
*Alternaria musae*	77.06%

### Morphological, cultural and physiological characterization

**Fig 1:**
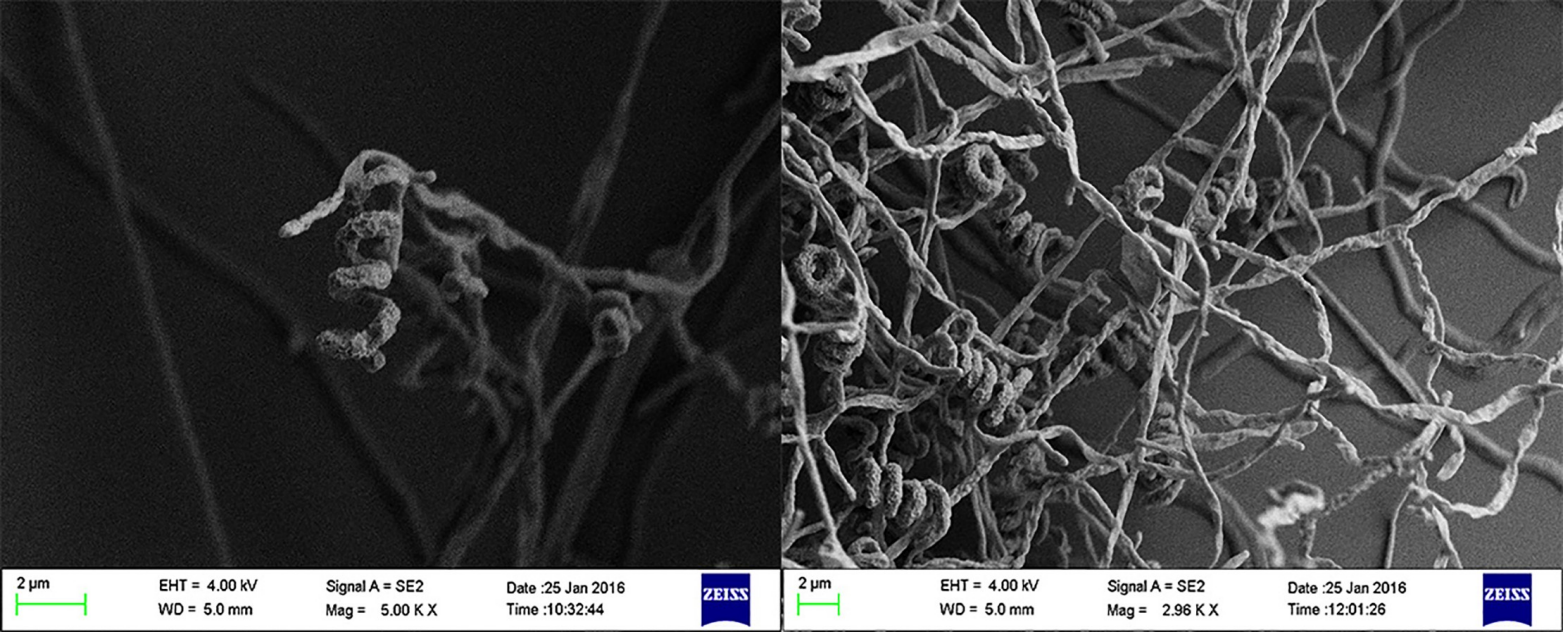
Scanning electron mirographs of strain 1–14.

Strain 1–14 exhibited typical morphological characteristics of the genus *Streptomyces* [37]. The cultural characteristics of the strain are recorded in the supplementary materials (S1 Table and S2 Fig). The strain grew well on ISP 7 medium, and no pigment was observed on any of the tested media. Scanning electron microscopic examination showed a branched mycelium with shorter air mycelium and compact, helical spore chains (Fig 1).

The physiological and biochemical characteristics of the strain 1–14 are recorded in the supplementary materials (S2 Table), as these assessments are indispensible tools for the classification and identification of actinomycetes. Growth of the strain occurred within a pH range of 6–8 with optimum growth at pH 7 and 28°C; furthermore, the strain exhibited salt tolerance at up to 5% NaCl. Strain 1–14 furthermore, the strain liquefaction, and nitrate reduction and exhibited positive responses for the urease, coagulase and esterase tests. However, strain 1–14 did not hydrolyse cellulose nor produce H_2_S.

The utilization of diverse carbon sources by the strain indicated its wide carbon assimilation ability (S3 Table). The strain efficiently utilized carbon sources such as soluble starch, anhydrous lactose, D-galactose, _α_-Lactose and valine. Carbohydrate utilization plays an important role in the taxonomic characterization of actinomycetes [38].

### Identification strain 1–14 by 16S rDNA and phylogenetic analyses

**Fig 2:**
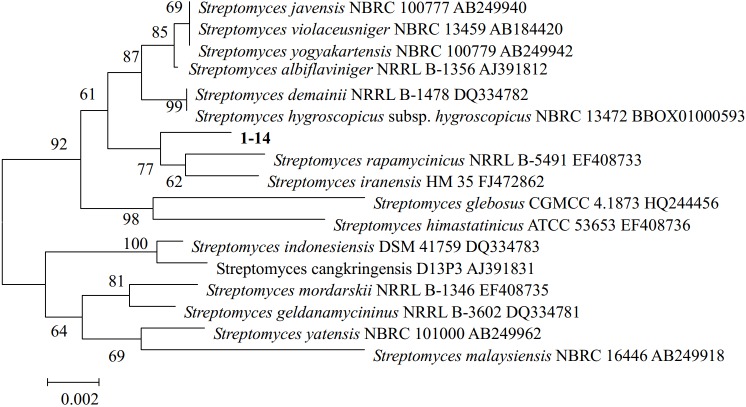
Phylogenetic tree of 16S rDNA gene sequences of strain 1–14.

A partial 16S rDNA gene fragment from strain 1–14 was sequenced and deposited in the GenBank database at the NCBI under the accession number MG897460. A phylogenetic tree with this sequence was constructed using the neighbor joining method with 1000 bootstrap replicates in MEGA version 5.1. Small genetic distance values were observed between the strain 1–14 sequence and those of *Streptomyces rapamycinicus* NRRL B-5491 and *Streptomyces iranensis* HM 35 sequences, indicating that they belong to the same genera (Fig 2). After comparing physiological and biochemical aspects with model organisms of the genus, strain 1–14 was assigned to *Streptomyces*.

### Propagation of *Streptomyces* sp. 1–14 in soil under simulated conditions

**Fig 3:**
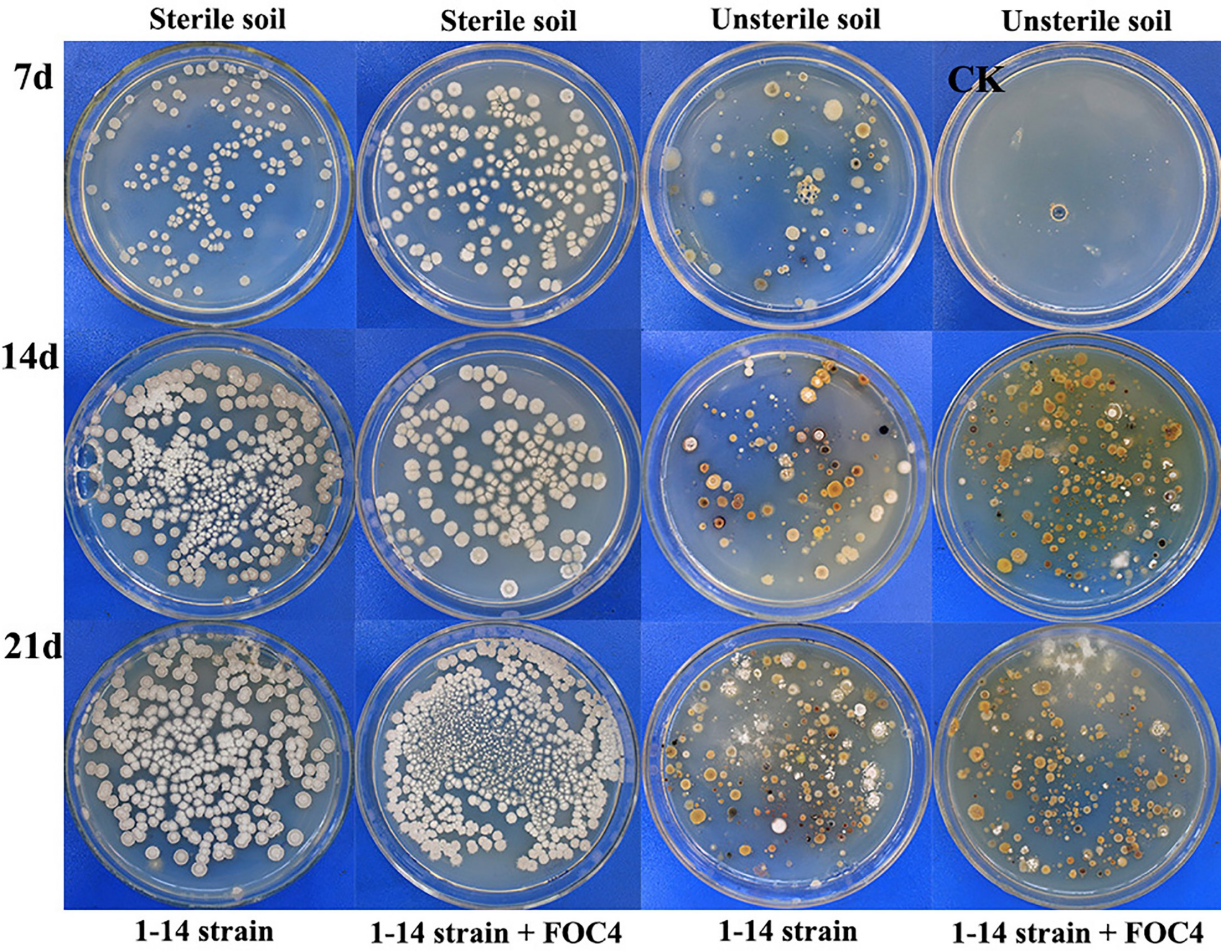
The colonization of antagonistic *Streptomycetes* sp. 1–14 in sterilized and unsterilized soils.

The propagation of *Streptomyces* sp. 1–14 in soil was simulated by the single inoculation of *Streptomyces* sp. 1–14 and its mixed inoculation with FOC4 (Table 3 and Fig 3). For both the single and mixed inoculations, the *J* values of the natural soil were small, while those of the sterilized soil were 100%. The *Streptomyces* sp. 1–14 populations in the sterilized soil were significantly larger than those in the unsterilized treatments. Increases in the *Streptomyces* population were inhibited by indigenous microorganisms in the soil. In the sterilized treatments, the *Streptomyces* sp. 1–14 population in the soil on the 21st day was larger that on 7th day, for both the single inoculation and the mixed inoculation with FOC4. Therefore, the multiplication and survival of *Streptomyces* sp. 1–14 in the soil was not significantly influenced by FOC4. When *Streptomyces* sp. 1–14 was inoculated alone in sterilized soil, its population increased before decreasing, which could be associated with changes in the soil moisture, nutrients, temperature or other environmental conditions.

**Table 3:** Population of soil *Streptomycetes* sp. 1–14 in treatments inoculated alone and with FOC4.

Treatment		Treatment time									
		7 d 14 d 21 d									
		Quantity(cfu/g)			*J* (%)	Quantity(cfu/g)		*J* (%)	Quantity(cfu/g)		*J* (%)
		∑*A*	*Q*			∑*A*	*Q*		∑*A*	*Q*	
**1–14**	**Unsterile soil**	2.70×106	3.40×105		12.59	2.40×106	2.40×105	10.00	2.36×106	4.38×105	18.56
	**Sterile soil**	1.00×105	1.00×105		100	3.14×106	3.14×106	100	3.12×106	3.12×106	100
**1–14+FOC4**	**Unsterile soil**	4.74×106	6.79×105		14.32	2.88×106	5.40×105	18.75	3.36×106	5.60×105	16.67
	**Sterile soil**	1.80×105	1.80×105		100	2.52×106	2.52×106	100	3.98×106	3.98×106	100

### Screening the most effective factors by Plackett-Burman design

The design of the Plackett-Burman test is shown in S5 Table. The effect of each factor on the response value *Y* (antibacterial activity) is listed in Table 4, and the following regression equation was obtained:

$$\begin{array}{l} Y=23.42583+3.25917X_{1}-2.28583X_{2}+7.7625X_{3}-1.01917X_{4}+2.70250X_{5}\\ \;+1.67417X_{6}+2.38583X_{7}-2.81750X_{8}-4.42083X_{9} \end{array}$$

The decision coefficient, R^2^, of the regression equation, R^2^, was 99.33%, indicating that the regression equation was suitable for the analysis and prediction of changes in the antibacterial activity of the crude extract during fermentation. The P-values of glucose, CaCl_2_•2H_2_O, temperature and the inoculation amount were less than 0.05 (Table 4), indicating that these four factors are key factors. The t-value indicates the positive and negative effects of each factor. A “+” indicates that the factor has a positive impact on antibacterial activity; otherwise, the factor has a negative impact. Table 4 shows that glucose and CaCl_2_•2H_2_O had positive effects on the activities of the fermentation products, while the temperature and the inoculation amount had negative effects.

**Table 4:** Analysis of the data generated by the Plackett–Burman design.

Coded variable	Variable	DF	Parameter estimate	t-value	P> |t|	Significance
Intercept	Constant	1	23.42583	36.69	0.0007	***
X_1_	Glucose	1	3.25917	5.11	0.0363	*
X_2_	Soy flour	1	-2.28583	-3.58	0.0699	
X_3_	CaCl_2_·2H_2_O	1	7.76250	12.16	0.0067	**
X_4_	NaH_2_PO_4_	1	-1.01917	-1.60	0.2515	
X_5_	MgSO_4_	1	2.70250	4.23	0.0515	
X_6_	K_2_HPO_4_	1	1.67417	2.62	0.1198	
X_7_	pH	1	2.38583	3.74	0.0647	
X_8_	Temperature	1	-2.81750	-4.41	0.0477	*
X_9_	The inoculation amount	1	-4.42083	-6.92	0.0202	*

Note: X_1_ ~ X_9_ represent various impact factors.

* Significant at the 0.05 level

** significant at the 0.01 level

*** significant at the 0.001 level.

#### Identifying the approximate ranges of optimal fermentation conditions for the key factors by determining the path of steepest ascent

From the Placket-Burman analysis, the most effective factors that significantly impact antibacterial activity were identified and subjected to the steepest ascent test. As seen in Table 1, the greatest antimicrobial activity was observed for the fifth group of experiments; thus, the fifth sets of tests were used as the central point of the response surface.

#### Determination of the best fermentation conditions using BBD

A four-factor and three-level BBD was used to analyze the interactions between the four factors to identify the best fermentation conditions. The design and results are shown in S7 Table. The response of the antibacterial rate was studied, and the resultant second-order polynomial equation was as follows:

$$\begin{array}{l} Y=54.79+3.23X_{1}+1.59X_{2}-0.066X_{3}-1.00X_{4}+2.35X_{1}X_{2}-0.15X_{1}X_{3}-0.095X_{1}X_{4}\\ \;+0.24X_{2}X_{3}-0.38X_{2}X_{4}+1.72X_{3}X_{4}-4.02X_{1}{}^{2}-4.72X_{2}{}^{2}-4.31X_{3}{}^{2}-2.66X_{4}{}^{2} \end{array}$$

where Y is the predicted value of the FOC4 inhibition rate, X_1_ is glucose, X_2_ is CaCl_2_•2H_2_O, X_3_ is temperature, and X_4_ is the inoculation amount.

The statistical significance of the fitted model was evaluated by ANOVA (Table 5). For the regression model, F = 53.94> F0.01 (5, 7) = 7.46 and P <0.0001, reaching the limit level, indicating that the regression equation was very good. The F-value of the lack-of-fit model was 0.39 <F0.01 (3, 4) = 16.7 and P was 0.8961> 0.05, which indicated that the actual value of the experiment was consistent with the predicted value of the model. Therefore, the non-test factors for the test results are not significant, and the model could be used. The coefficient of determination (R^2^) was 0.9818, and the model could be used to effectively predict the response. The adjusted coefficient of determination (R^2^) was 0.9636, which further showed the reliability of the model. The coefficient of variation was 1.65%, which indicated that the equation of this model reflects the test value. Therefore, the regression model was believed to accurately and reliably predict and analyze the antibacterial activity of the actinomycete strain 1–14.

**Table 5:** Results of the ANOVA for the regression equation.

Source	DF	SS	MS	F-value	P-value Prob>F
Model	14	476.94	34.07	53.94	<0.0001
X_1_- Glucose	1	125.26	125.26	198.33	<0.0001
X_2_-CaCl_2_·2H_2_O	1	30.24	30.24	47.88	<0.0001
X_3_- Temperature	1	0.052	0.052	0.082	0.7783
X_4_-The inoculation amount	1	12.06	12.06	19.10	0.0006
X_1_ X_2_	1	22.00	22.00	34.83	< 0.0001
X_1_ X_3_	1	0.093	0.093	0.15	0.7069
X_1_ X_4_	1	0.036	0.036	0.057	0.8145
X_2_ X_3_	1	0.24	0.24	0.37	0.5515
X_2_ X_4_	1	0.58	0.58	0.91	0.3551
X_3_ X_4_	1	11.87	11.87	18.79	0.0007
X_1_^2^	1	104.76	104.76	165.88	< 0.0001
X_2_^2^	1	144.59	144.59	228.94	< 0.0001
X_3_^2^	1	120.57	120.57	190.91	< 0.0001
X_4_^2^	1	45.94	45.94	72.74	< 0.0001
Residual	14	8.84	0.63		
Lack of Fit	10	4.37	0.44	0.39	0.8961
Pure Error	4	4.47	1.12		
Cor Total	28	485.78			
Coefficient of determination R^2^ = 0.9818		Adjusted coefficient of determination R^2^ = 0.9636		Mean = 48.28	Coefficient of variation (CV) = 1.65%

Note: P> 0.05, insignificant difference; P <0.05, significant difference; P <0.01, significant difference.

The binomial coefficients X_1_^2^, X_2_^2^, X_3_^2^ and X_4_^2^ of the regression equation were highly significant, indicating that the change of the response value (antibacterial rate) was complex (S8 Table). The effect of the factors on the antibacterial activity of strain 1–14 was not a simple linear relationship but a significant surface effect. The coefficient of the quadratic term was negative, and the equation had a maximum value; thus, the response surface opened facing downwards. The P-values of the monomial coefficients X_1_, X_2_ and X_4_ were less than 0.05, indicating that the linear effects of strain 1–14 were significant. The P-value of X_3_ was greater than 0.05, indicating an insignificant linear effect. The interactions between X_1_ X_2_ and X_3_ X_4_ were significantly, indicating that there is an interaction between the two factors exists.

#### Response surface and contour plots

**Fig 4:**
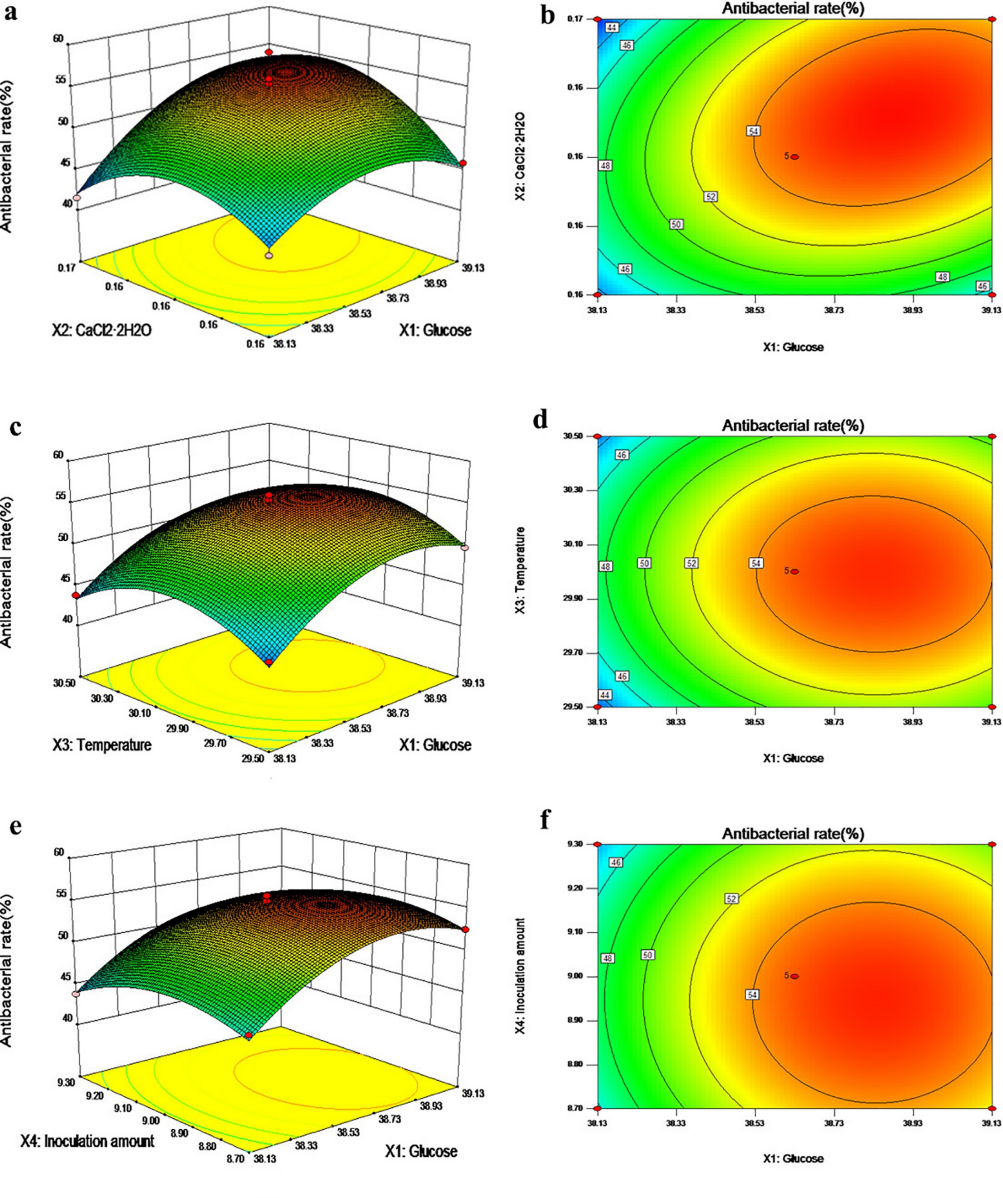
Response surface plots (3D) and contour plots (2D) showing the individual and interactive effects of variables on the antibacterial activity of actinomycete strain 1–14. (a, b) Effects of glucose and CaCl_2_•2H_2_O on antimicrobial activity; (c, d) effects of glucose and temperature on antimicrobial activity; (e, f) effects of glucose and the inoculation amount on antimicrobial activity.

**Fig 5:**
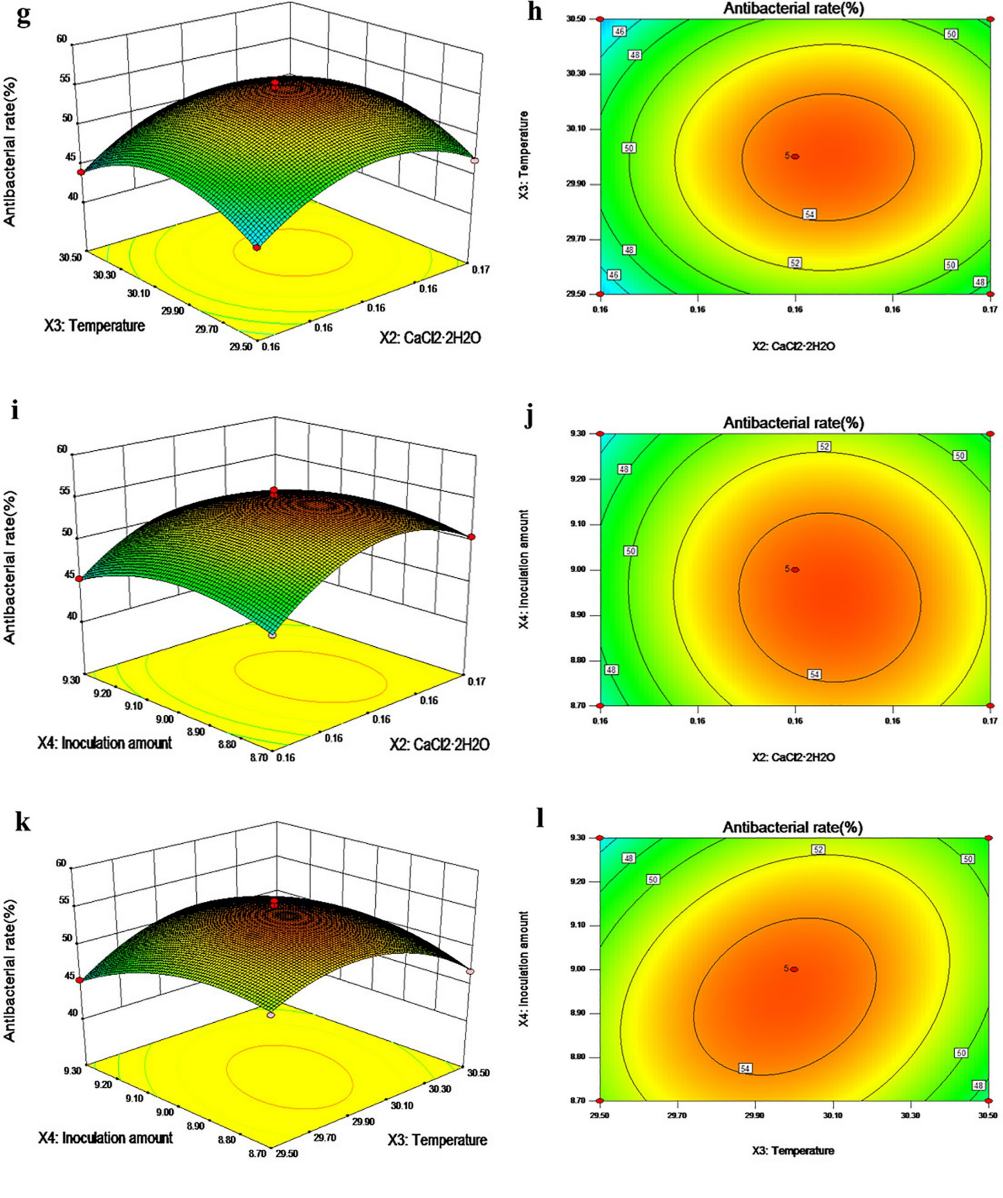
Response surface plots (3D) and contour plots (2D) showing the individual and interactive effects of variables on the antibacterial activity of actinomycete strain 1–14. (g, h) Effects of CaCl_2_•2H_2_O and temperature on antimicrobial activity; (i, j) effects of CaCl_2_•2H_2_O and the inoculation amount on antimicrobial activity; (k, l) effects of temperature and the inoculation amount on antimicrobial activity.

The response value analysis and contours of the antimicrobial activity with two independent variables as coordinates are shown in Fig 4 and Fig 5. From the contours, we found that the interactions between glucose and CaCl_2_•2H_2_O, glucose and the inoculation amount, CaCl_2_•2H_2_O and the inoculation amount, and temperature and the inoculation amount were elliptical, indicating that the effects of the interactions between each of the two factors are significant. The glucose and temperature and CaCl_2_•2H_2_O and temperature contour lines were round, indicating a lack of significance. The 3-Dimensional RSM plots clearly indicated that the maximum antibacterial activity should occur with medium levels of glucose, CaCl_2_•2H_2_O, temperature and the inoculation amount. With the help of numerical optimization, the quadratic model predicted that the optimal values of the test factors were: glucose, 38.877 g/L; CaCl_2_•2H_2_O, 0.161 g/L; temperature, 29.97°C; and the inoculation amount, 8.93%. Furthermore, with these values, the maximum inhibitory rate achieved should be 55.94%

### Validation of the test results

To test the reliability of the models in predicting optimal responses and in accordance with the optimization results obtained from RSM with the desirability function, verification experiments were carried out at the optimal levels. The results (Table 6) indicated that the antibacterial activity was 56.13% at the selected optimum conditions, which was 12.33% higher than that before optimization (43.80%); the observed value was close to the predicted value (55.94%). The predicted results matched well with the experimental results obtained using optimal conditions, which validating the RSM models with good correlation.

**Table 6:** Comparison of the results obtained with the original and optimal conditions with strain 1–14.

Content	Antimicrobial activity against FOC4
Optimal condition	56.13%
Original condition	43.80%

### Stability of the crude fermented extract

**Fig 6:**
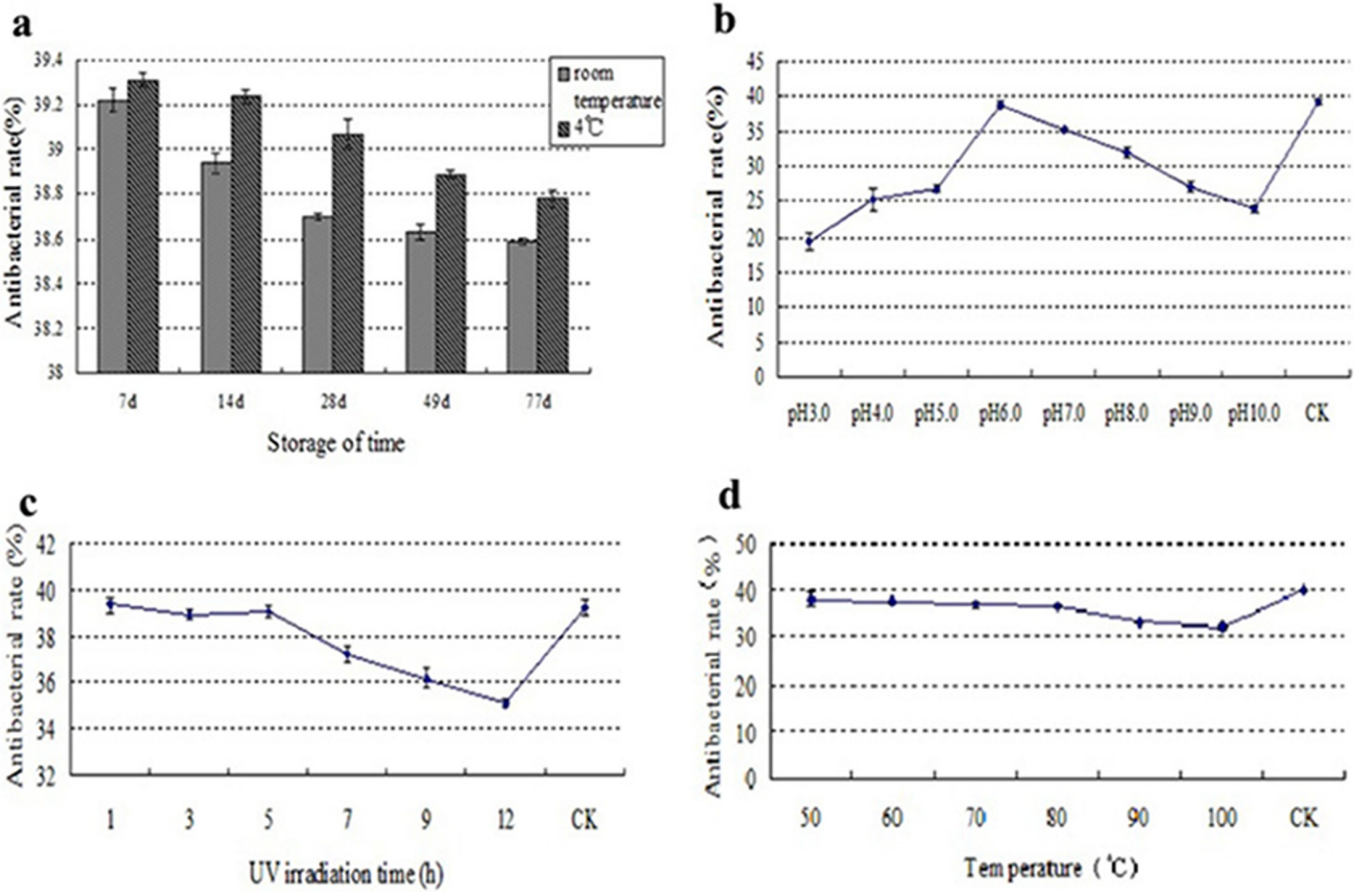
Stability of the crude fermented extract. (a) Stability during storage over time; (b) pH stability; (c) UV irradiation stability; (d) thermal stability.

The storage stability of the crude extract was good (Fig 6A). Storage at 4°C was better than storage at room temperature, as 4°C is more conducive to maintaining high antibacterial activity. Under strongly acidic or strongly alkaline conditions, the antibacterial activity of the crude fermented extract was low (Fig 6B). At pH values of 3.0 and 10.0, the antibacterial activity was the lowest, indicating that the crude fermented extract was sensitive to strong acids and strong bases. Therefore, the pH value should be closely monitored to reduce the loss of active antimicrobial substance(s). The antibacterial activity of strain 1–14 was stable after 1 to 5 h of UV irradiation, but after 5 h, the antibacterial activity decreased (Fig 6C), indicating that UV irradiation has a negative effect on the active antibacterial substance(s). The crude extract was very stable at high temperatures (Fig 6D); while the crude fermented extracts exhibited decreased anti-FOC4 activity with increases in temperature, the decreases were not substantial.

## Discussion

*Streptomyces* species are renowned sources of novel secondary metabolites that have a range of biological activities such as antimicrobial and anticancer activities [6]. The isolation of new microbial species from unexplored and extreme habitats is one of the most efficient strategies for the mining potential microbial compounds. To date, no reports are available regarding the actinomycetes in cassava rhizosphere soil. Therefore, the present study aimed to isolate and screen anti-FOC4 *Streptomyces* species from cassava rhizosphere soil and optimized culture conditions for secondary metabolites production. It was isolated 63 morphologically distinct actinomycete strains from Cassava rhizosphere soil. Thirteen actinomycetes exhibited antibacterial activity against FOC4, and a strain 1–14 among them showed broad-spectrum antibacterial activity against pathogenic fungi. Morphological studies and 16S rDNA analysis of strain 1–14 showed that it belonged to *Streptomyces*; thus, it was named *Streptomyces* sp. 1–14. Different species in this genus, i.e., *Streptomyces noursei* [7], *Streptomyces rimosus* [39], *Streptomyces luozhongensis* [40], have been reported to have anti-FOC activity.

The propagation of *Streptomyces* sp. 1–14 in soil under simulated conditions showed that the populations of *Streptomyces* sp. 1–14 in sterilized soil were significantly higher than those in unsterilized treatments, likely because the *Streptomyces* sp. 1–14 populations were inhibited by indigenous microorganisms in the unsterilized soils. In the sterilized treatments, the multiplication and survival of *Streptomyces* sp. 1–14 in soil were not significantly influenced by FOC4. When *Streptomyces* sp. 1–14 was inoculated alone in sterilized soil, its population increased and then decreased, a trend that was associated with changes in the soil moisture, nutrients, temperature and other environmental conditions [30].

Most of the metabolites produced by *Streptomyces* are extracellular in nature and nave potent antimicrobial activities [41]. Microbial fermentation is a complex, non-linear, unstructured process. Minor variations in the composition of fermentation media and conditions can influence the yields of certain compounds and lead to changes in the metabolic profile of a strain [42–43]. Determining the optimal fermentation conditions is difficult. To obtain the best fermentation conditions, designing an experiment using a reasonable experimental method is necessary. The composition of the fermentation medium and the culture conditions can be optimized by RSM to improve the production of secondary metabolites, thereby promoting the discovery of novel natural active ingredients [44–46]. In this study, RSM was used to optimize the fermentation conditions. First, Plackett-Burman design was used to identify the main factors affecting metabolite production and anti-FOC4 activity, which were determined to be glucose, CaCl_2_•2H_2_O, temperature and the inoculation amount. Then, the path of steepest ascent design was used to determine the approximate ranges of the optimal fermentation conditions for the key factors, which were used as the central point of the response surface (glucose, 38.63 g/L; CaCl_2_•2H_2_O, 0.16 g/L; temperature, 30°C; and the inoculation amount, 9.0%). Next, Box-Behnken design was used to establish the fermentation model and to analyze the optimal values of the fermentation conditions (glucose, 38.877 g/L; CaCl_2_•2H_2_O, 0.161 g/L; temperature, 29.97°C; and the inoculation amount, 8.93%). To test the reliability of the models in predicting optimum responses, the test result was validated. The antibacterial activity of strain 1–14 was 56.13%, which was 12.33% higher than the antibacterial activity before optimization (43.80%) and was close to the predicted value (55.94%). The predicted results matched well with the experimental results obtained using the optimal conditions, which validating the RSM models with good correlation. The RSM model had a significant effect on the production of bioactive metabolites by *Streptomyces* sp. 1–14. A substantial increase in the anti-FOC4 activity of the crude fermentation extract occurred, and the reasons for this increase are as follows: 1) the optimized of fermentation conditions improved the production of active antibacterial ingredients and 2) the metabolic profile of the strain changed, resulting in more active antibacterial ingredients. Further study is required to determine the specific reason.

A rapidly metabolizable source of carbon has a negative effect on biosynthesis (due to catabolic repression or the "glucose effect") [47]. However, an increase in the amount of glucose had a significant effect and improved the metabolic activity of strain 1–14. Many other researchers have found the glucose concentration to be a significant factor in the production of active antibacterial substances, and showing increased antibacterial activity at higher glucose concentrations [48–49]. For example, in *Streptomyces griseocarneus* and *Streptomyces padanus* PMS-702, the best carbon source for the production of antifungal polyenes was glucose [17]. Increasing the amount of CaCl_2_•2H_2_O also had a significant effect and improved the metabolic activity of strain 1–14. Calcium is not part of the cell composition, but it can control cell permeability; hence, calcium has an important impact on the growth of microorganisms [50]. Temperature is a physiological parameter that alters the fermentation process. Fermentation can affect the regulation of ATP, which, in turn, influences the regulation of metabolic pathways and cell-wall synthesis, thereby causing diverse metabolic effects and generating a variety of products [51–52]. Due to the slow growth of actinomycetes, the biological activity of these organisms may be affected at high or low temperatures [52]. Therefore, CaCl_2_ and temperature influenced metabolite production significantly [53–55]. A low inoculation amount may lead to insufficient biomass accumulation, causing reduced product formation. Conversely, a high inoculation amount can produce too much biomass, leading to poor product formation [55]. The optimal inoculation amount determined for strain 1–14 was 8.93%.

Antibacterial activity was significantly reduced under meta-acidic or meta-alkaline conditions. The antimicrobial activity after 12 h of UV irradiation was 10.76% lower than that after 1 h. These decreases in antibacterial activity may have been due to decomposition or structural changes of the active ingredient under UV irradiation or acidic/basic conditions, thereby altering the activity. Therefore, during fermentation and storage, pH, light, storage temperature, etc., should be monitored as closely as possible to reduce the formation of fermentation products and to prevent their inactivation, which can both lead to reduced levels of the active ingredient(s) [36, 56–58].

Regardless of the medium and optimized of the culture conditions, the anti-FOC4 ability of strain 1–14 in natural environments should be seriously considered. In this respect, the present study is useful for further investigations of the industrial production of anti-FOC4 substances as well as the purification of anti-FOC4 compounds. With a long-term goal of developing an effective method for controlling FOC4, further studies will focus on identifying the active anti-FOC4 compounds and elucidating the FOC4 inhibition mechanism of *Streptomyces* sp. 1–14.

## Conclusions

Sixty-three morphologically distinct actinomycete strains were isolated from the rhizosphere soil of cassava samples. Among them, strain 1–14 showed broad-spectrum antibacterial activity against pathogenic fungi. Morphological studies and 16S rDNA analysis showed that strain 1–14 belongs to *Streptomyces*; thus, it was named *Streptomyces* sp. 1–14. Through the propagation of *Streptomyces* sp. 1–14 in soil under simulated conditions, FOC4 was found to have no significant influence on the multiplication and survival of *Streptomyces* sp. 1–14; however, indigenous microorganisms in the soil significantly influenced the *Streptomyces* sp.1-14 populations. To achieve maximum metabolite production, fermentation of *Streptomyces* sp. 1–14 was optimized through response surface methodology. The optimization resulted in antibacterial activity of 56.13% against FOC4, which was 12.33% higher than that before optimization (43.80%). The results obtained using response surface methodology to optimize the fermentation medium had a significant effect on the production of bioactive metabolites by *Streptomyces* sp. 1–14. In addition, its antibacterial activity was significantly reduced under meta-acidic, meta-alkaline or UV irradiation conditions.

## Supporting information

S1 Fig Antibacterial activity of 1–14 strain against pathogens. (TIF)

S2 Fig The colonies characteristic of the 1–14 strain on different Medias. (TIF)

S1 Table The cultural characteristics of the strain 1–14 in six different Medias. (DOC)

S2 Table Results of physiological and biochemical characterization. (DOC)

S3 Table Carbon and nitrogen source utilization. (DOC)

S4 Table Through Plackett–Burman design to determined the levels of factors. (DOC)

S5 Table Experimental designs and results of Plackett–Burman design. (DOC)

S6 Table Coded and actual values of the factors tested in the BBD. (DOC)

S7 Table The matrix and data of the BBD experiment. (DOC)

S8 Table Regression coefficients and their significance in the quadratic model. (DOC)
