# Supplementary figures and images for: Optimization of fermentation conditions through response surface methodology for enhanced antibacterial metabolite production by Streptomyces sp. 1-14 from cassava rhizosphere
Source: PLoS One. 2018 Nov 14;13(11):e0206497. doi: 10.1371/journal.pone.0206497 (PMC6241123; doi:10.1371/journal.pone.0206497)

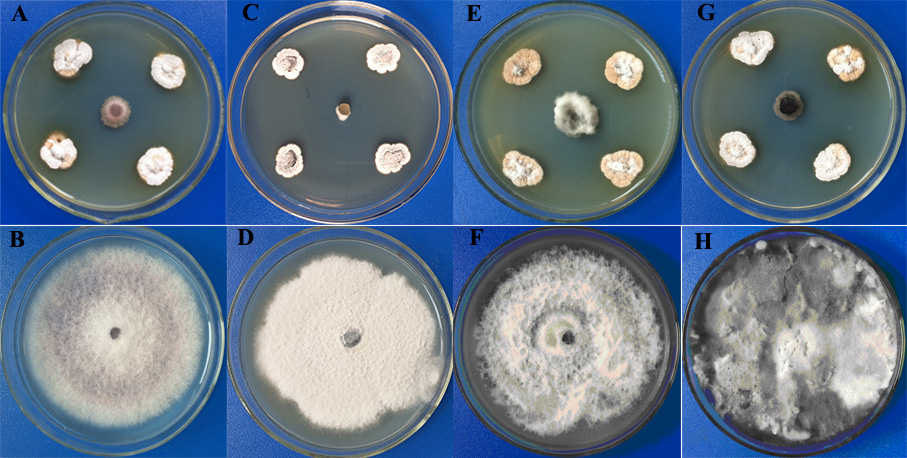

Supplement: S1 Fig — (TIF) [file pone.0206497.s001.tif]

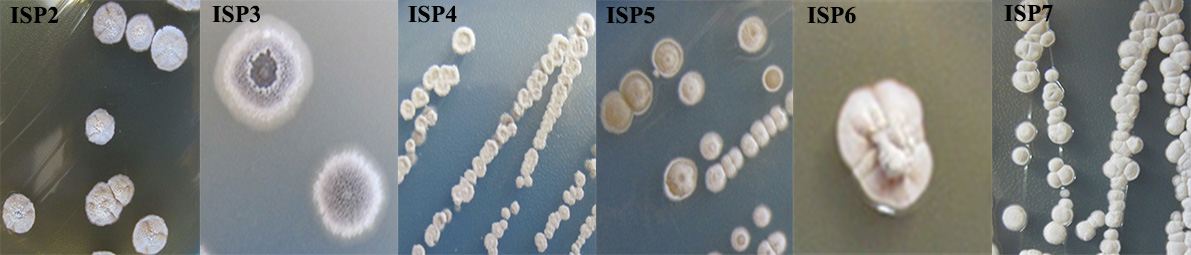

Supplement: S2 Fig — (TIF) [file pone.0206497.s002.tif]
