# Supplementary material for: Optimization of fermentation conditions through response surface methodology for enhanced antibacterial metabolite production by Streptomyces sp. 1-14 from cassava rhizosphere
Source: PLoS One. 2018 Nov 14;13(11):e0206497. doi: 10.1371/journal.pone.0206497 (PMC6241123; doi:10.1371/journal.pone.0206497)
Supplement: S1 Table — (DOC) [file pone.0206497.s003.doc]

**S1 Table. The cultural characteristics of the strain 1-14 in six different Medias**

| **Strain** | **Medium** | **Aerial mycelium** | **Spore colour** | **Kiene mycelium** | **Soluble pigment** | **Growth status** |
| --- | --- | --- | --- | --- | --- | --- |
|  | ISP2 | White | White | Khaki | None | Moderate |
|  | ISP3 | Creamy white | Grey | Pale yellow | None | Moderate |
| 1-14 | ISP4 | Pale grey | White | Creamy white | None | Good |
|  | ISP5 | snow | White | Pale yellow | None | Moderate |
|  | ISP6 | snow | White | Dark yellow | None | Poor |
|  | ISP7 | snow | White | Brown | None | Good |
