# Supplementary material for: Optimization of fermentation conditions through response surface methodology for enhanced antibacterial metabolite production by Streptomyces sp. 1-14 from cassava rhizosphere
Source: PLoS One. 2018 Nov 14;13(11):e0206497. doi: 10.1371/journal.pone.0206497 (PMC6241123; doi:10.1371/journal.pone.0206497)
Supplement: S2 Table — (DOC) [file pone.0206497.s004.doc]

**S2 Table. Results of physiological and biochemical characterization**

| **Strain** | **Starch hydrolysis** | **Gelatin liquefaction** | **Cellulose hydrolysis** | **Nitrate reduction** | **H_2_S** | **Urease test** | **Esterase test** | |
| --- | --- | --- | --- | --- | --- | --- | --- | --- |
|  |  |  |  |  |  |  | **Twain 20** | **Twain 80** |
| 1-14 | + | + | - | + | - | + | + | + |

Note: In the test items, the "+" means positive, "-" indicates negative.
