# Supplementary material for: Optimization of fermentation conditions through response surface methodology for enhanced antibacterial metabolite production by Streptomyces sp. 1-14 from cassava rhizosphere
Source: PLoS One. 2018 Nov 14;13(11):e0206497. doi: 10.1371/journal.pone.0206497 (PMC6241123; doi:10.1371/journal.pone.0206497)
Supplement: S3 Table — (DOC) [file pone.0206497.s005.doc]

**S3 Table. C**arbon and nitrogen source utilization

| **Carbon test items** | **Result** |  | **Nitrogen test items** | **Result** |
| --- | --- | --- | --- | --- |
|  |
| CK | - |  | CK | - |
| L-Rhamnose | +++ |  | Glycine | + |
| D- Ribose | +++ |  | Phenylalanine | ++ |
| Melezitose | +++ |  | **Methionine** | ++ |
| Lactose anhydrous | +++ |  | **Hydroxyproline** | ++ |
| **Inositol** | + |  | **Arginine** | - |
| D-Mannitol | +++ |  | Phenylalanate | + |
| D-Xylose | +++ |  | **Histidine** | + |
| L-Arabinose | +++ |  | **Ammonium oxalate** | - |
| Salicin | +++ |  | **Cysteine** | ++ |
| D- Mannose | +++ |  | Ammonium sulphate | + |
| D- Galactose | +++ |  | Ammonium nitrate | ++ |
| D- Trehalose dihydrate | ++ |  | Ammonium acetate | - |
| Starch | +++ |  | **Serine** | ++ |
| Sorbitol | +++ |  | Ammonium molybdate tetrahydrate | - |
| D-Fructose | +++ |  | **Valine** | +++ |
| α- Lactose | +++ |  | **Glycine** | + |

Note: "+++" is indicates growth in carbon or nitrogen source in good, "++" is indicates growth in carbon or nitrogen source in general, "+" is indicates growth in carbon or nitrogen source is weak, "-" is indicates no growth in carbon or nitrogen sources.
