# Supplementary material for: Optimization of fermentation conditions through response surface methodology for enhanced antibacterial metabolite production by Streptomyces sp. 1-14 from cassava rhizosphere
Source: PLoS One. 2018 Nov 14;13(11):e0206497. doi: 10.1371/journal.pone.0206497 (PMC6241123; doi:10.1371/journal.pone.0206497)
Supplement: S4 Table — (DOC) [file pone.0206497.s006.doc]

**S4 Table. Through Plackett–Burman design to determined the levels of factors**

| **Number** | **Factors** | **level** | |
| --- | --- | --- | --- |
|  |  | **-1** | **1** |
| X_1_ | Glucose (g/L) | 30.00 | 37.50 |
| X_2_ | Soy flour (g/L) | 20.00 | 25.00 |
| X_3_ | CaCl_2_·2H_2_O(g/L) | 0.1 | 0.13 |
| X_4_ | NaH_2_PO_4_(g/L) | 0.5 | 0.63 |
| X_5_ | MgSO_4_ (g/L) | 0.2 | 0.25 |
| X_6_ | K_2_HPO_4_(g/L) | 0.5 | 0.63 |
| X_7_ | pH | 8 | 10 |
| X_8_ | Temperature (°C) | 31 | 38 |
| X_9_ | Inoculation amount (%) | 10 | 12.5 |

Note: X_1_-X_9_ represent various impact factors; "1" and "-1" represent two different levels.
