# Supplementary material for: Optimization of fermentation conditions through response surface methodology for enhanced antibacterial metabolite production by Streptomyces sp. 1-14 from cassava rhizosphere
Source: PLoS One. 2018 Nov 14;13(11):e0206497. doi: 10.1371/journal.pone.0206497 (PMC6241123; doi:10.1371/journal.pone.0206497)
Supplement: S5 Table — (DOC) [file pone.0206497.s007.doc]

**S5 Table. Experimental design and results of Plackett–Burman design**

| **Run** | **Variable** | | | | | | | | | **Antibacterial activity (%)** |
| --- | --- | --- | --- | --- | --- | --- | --- | --- | --- | --- |
|  | **X_1_** | **X_2_** | **X_3_** | **X_4_** | **X_5_** | **X_6_** | **X_7_** | **X_8_** | **X_9_** |  |
| 1 | - 1 | 1 | 1 | - 1 | 1 | 1 | 1 | - 1 | - 1 | 40.49 |
| 2 | 1 | - 1 | 1 | 1 | - 1 | 1 | 1 | 1 | - 1 | 39.94 |
| 3 | - 1 | 1 | 1 | 1 | - 1 | - 1 | - 1 | 1 | - 1 | 18.20 |
| 4 | 1 | 1 | - 1 | 1 | 1 | 1 | - 1 | - 1 | - 1 | 25.02 |
| 5 | 1 | - 1 | - 1 | - 1 | 1 | - 1 | 1 | 1 | - 1 | 25.98 |
| 6 | - 1 | - 1 | - 1 | 1 | - 1 | 1 | 1 | - 1 | 1 | 12.16 |
| 7 | - 1 | - 1 | - 1 | - 1 | - 1 | - 1 | - 1 | - 1 | - 1 | 17.45 |
| 8 | 1 | 1 | 1 | - 1 | - 1 | - 1 | 1 | - 1 | 1 | 29.76 |
| 9 | - 1 | 1 | - 1 | 1 | 1 | - 1 | 1 | 1 | 1 | 6.54 |
| 10 | 1 | 1 | - 1 | - 1 | - 1 | 1 | - 1 | 1 | 1 | 6.83 |
| 11 | 1 | - 1 | 1 | 1 | 1 | - 1 | - 1 | - 1 | 1 | 32.58 |
| 12 | - 1 | - 1 | 1 | - 1 | 1 | 1 | - 1 | 1 | 1 | 26.16 |

Note: X_1_ ~ X_9_ represent various impact factors; "1" and "-1" represent two different levels; 1 to 12 represent 12 different sets of culture conditions.
