# Supplementary material for: Optimization of fermentation conditions through response surface methodology for enhanced antibacterial metabolite production by Streptomyces sp. 1-14 from cassava rhizosphere
Source: PLoS One. 2018 Nov 14;13(11):e0206497. doi: 10.1371/journal.pone.0206497 (PMC6241123; doi:10.1371/journal.pone.0206497)
Supplement: S6 Table — (DOC) [file pone.0206497.s008.doc]

**S6 Table. Coded and actual values of the factors tested in the BBD**

| **Level** | **Factors** | | | |
| --- | --- | --- | --- | --- |
|  | **Glucose (g/L)** | **CaCl_2_·2H_2_O (g/L)** | **Temperature (°C)** | **Inoculation amount (%)** |
| -1 | 38.13 | 0.155 | 29.5 | 8.7 |
| 0 | 38.63 | 0.160 | 30.0 | 9.0 |
| 1 | 39.13 | 0.165 | 30.5 | 9.3 |
