# Supplementary material for: Optimization of fermentation conditions through response surface methodology for enhanced antibacterial metabolite production by Streptomyces sp. 1-14 from cassava rhizosphere
Source: PLoS One. 2018 Nov 14;13(11):e0206497. doi: 10.1371/journal.pone.0206497 (PMC6241123; doi:10.1371/journal.pone.0206497)
Supplement: S7 Table — (DOC) [file pone.0206497.s009.doc]

**S7 Table.** The matrix and data of the BBD experiment

| **Run** | **Variable** | | | | **Antibacterial activity (%)** | | |
| --- | --- | --- | --- | --- | --- | --- | --- |
|  | **Glucose (g/L)** | **CaCl_2_·2H_2_O**  **(g/L)** | **Temperature (℃)** | **Inoculation amount (%)** | ***Y* (Test value)** | ***Y* (Fit value)** | **Fitting error** |
| 1 | 0 | 0 | 1 | -1 | 46.77 | 47.03 | -0.26 |
| 2 | 0 | 1 | 0 | -1 | 50.49 | 50.37 | 0.12 |
| 3 | 0 | 0 | 0 | 0 | 55.38 | 54.79 | 0.59 |
| 4 | 0 | 1 | 1 | 0 | 47.16 | 47.52 | -0.36 |
| 5 | 0 | 0 | 0 | 0 | 54.78 | 54.79 | -0.01 |
| 6 | 0 | 1 | 0 | 1 | 47.94 | 47.61 | 0.33 |
| 7 | -1 | 0 | -1 | 0 | 43.71 | 43.14 | 0.57 |
| 8 | 1 | 0 | -1 | 0 | 49.67 | 49.90 | -0.23 |
| 9 | -1 | - 1 | 0 | 0 | 42.73 | 43.57 | -0.84 |
| 10 | 1 | 0 | 0 | -1 | 52.49 | 52.43 | 0.056 |
| 11 | 1 | 0 | 0 | 1 | 49.48 | 50.24 | -0.76 |
| 12 | 0 | 0 | -1 | -1 | 50.19 | 50.60 | -0.41 |
| 13 | 0 | 0 | 0 | 0 | 55.99 | 54.79 | 1.20 |
| 14 | 1 | - 1 | 0 | 0 | 45.84 | 45.34 | 0.50 |
| 15 | 0 | 0 | 0 | 0 | 53.16 | 54.79 | -1.63 |
| 16 | 1 | 1 | 0 | 0 | 53.98 | 53.21 | 0.77 |
| 17 | 0 | 0 | 0 | 0 | 54.62 | 54.79 | -0.17 |
| 18 | 1 | 0 | 1 | 0 | 49.14 | 49.47 | -0.33 |
| 19 | 0 | 0 | -1 | 1 | 45.34 | 45.15 | 0.19 |
| 20 | 0 | -1 | 1 | 0 | 43.98 | 43.86 | 0.12 |
| 21 | 0 | -1 | 0 | -1 | 46.35 | 46.44 | -0.088 |
| 22 | 0 | -1 | -1 | 0 | 44.66 | 44.47 | 0.19 |
| 23 | 0 | 1 | -1 | 0 | 46.87 | 47.16 | -0.29 |
| 24 | 0 | -1 | 0 | 1 | 45.32 | 45.19 | 0.13 |
| 25 | -1 | 0 | 0 | 1 | 43.74 | 43.97 | -0.23 |
| 26 | -1 | 0 | 1 | 0 | 43.79 | 43.31 | 0.48 |
| 27 | 0 | 0 | 1 | 1 | 48.81 | 48.47 | 0.34 |
| 28 | -1 | 1 | 0 | 0 | 41.49 | 42.06 | -0.57 |
| 29 | -1 | 0 | 0 | -1 | 46.37 | 45.78 | 0.59 |
