# Supplementary material for: Optimization of fermentation conditions through response surface methodology for enhanced antibacterial metabolite production by Streptomyces sp. 1-14 from cassava rhizosphere
Source: PLoS One. 2018 Nov 14;13(11):e0206497. doi: 10.1371/journal.pone.0206497 (PMC6241123; doi:10.1371/journal.pone.0206497)
Supplement: S8 Table — (DOC) [file pone.0206497.s010.doc]

**Table S8** Regression coefficients and their significance in the quadratic model

| Factor | Coefficient | Standard error of coefficient | 95% CI  Low | 95% CI  High |
| --- | --- | --- | --- | --- |
| Intercept | 54.79 | 0.36 | 54.02 | 55.55 |
| X_1_- Glucose | 3.23 | 0.23 | 2.74 | 3.72 |
| X_2_-CaCl_2_·2H_2_O | 1.59 | 0.23 | 1.10 | 2.08 |
| X_3_- Temperature | -0.066 | 0.23 | -0.56 | 0.43 |
| X_4_-Inoculation amount | -1.00 | 0.23 | -1.49 | -0.51 |
| X_1_ X_2_ | 2.35 | 0.40 | 1.49 | 3.20 |
| X_1_ X_3_ | -0.15 | 0.40 | -1.00 | 0.70 |
| X_1_ X_4_ | -0.095 | 0.40 | -0.95 | 0.76 |
| X_2_ X_3_ | 0.24 | 0.40 | -0.61 | 1.09 |
| X_2_ X_4_ | -0.38 | 0.40 | -1.23 | 0.47 |
| X_3_ X_4_ | 1.72 | 0.40 | 0.87 | 2.57 |
| X_1_^2^ | -4.02 | 0.31 | -4.69 | -3.35 |
| X_2_^2^ | -4.72 | 0.31 | -5.39 | -4.05 |
| X_3_^2^ | -4.31 | 0.31 | -4.98 | -3.64 |
| X_4_^2^ | -2.66 | 0.31 | -3.33 | -1.99 |
